# Metagenomic and genomic analysis of heavy metal-tolerant and -resistant bacteria in resource islands in a semi-arid zone of the Colombian Caribbean

**DOI:** 10.1007/s11356-023-30253-w

**Published:** 2023-12-21

**Authors:** Andrea Carolina Herrera-Calderon, Leslie Leal, Jeimy Daniela Suárez-Bautista, Hillary Sharid Manotas-Viloria, Andrea Muñoz-García, Diego Franco, Nelson Enrique Arenas, Javier Vanegas

**Affiliations:** 1 Faculty of Sciences, Department of Biology, Universidad Antonio Nariño, Bogotá, Colombia; 2 Department of Biological and Environmental Sciences, Universidad Jorge Tadeo Lozano, Bogotá, Colombia; 3 Faculty of Health Sciences, Universidad Colegio Mayor de Cundinamarca, Bogotá, Colombia; 4 Faculty of Sciences, Pontificia Universidad Javeriana, Bogotá, Colombia; 5 Faculty of Biology, Institute of Environmental Sciences, Jagiellonian University, Kraków, Poland

**Keywords:** Metagenomics, Resource islands, Heavy metals, Resistance, Tolerance, *Bacillus velezensis*, *Cytobacillus gottheilii*

## Abstract

Bacteria from resource islands can adapt to different extreme conditions in semi-arid regions. We aimed to determine the potential resistance and tolerance to heavy metals from the bacterial community under the canopy of three resource islands in a semi-arid zone of the Colombian Caribbean. Total DNA was extracted from soil and through a metagenomics approach, we identified genes related to heavy metal tolerance and resistance under the influence of drought and humidity conditions, as well as the presence or absence of vegetation. We characterized the genomes of bacterial isolates cultivated in the presence of four heavy metals. The abundances of genes related to heavy metal resistance and tolerance were favored by soil moisture and the presence of vegetation. We observed a high abundance of resistance genes (60.4%) for Cu, Zn, and Ni, while 39.6% represented tolerance. These genes positively correlated with clay and silt content, and negatively correlated with sand content. Resistance and tolerance were associated with detoxification mechanisms involving oxidoreductase enzymes, metalloproteases, and hydrolases, as well as transmembrane proteins involved in metal transport such as efflux pumps and ion transmembrane transporters. The *Bacillus velezensis* C3-3 and *Cytobacillus gottheilii* T106 isolates showed resistance to 5 mM of Cd, Co, Mn, and Ni through detoxification genes associated with ABC pumps, metal transport proteins, ion antiporter proteins, and import systems, among others. Overall, these findings highlight the potential of bacteria from resource islands in bioremediation processes of soils contaminated with heavy metals.

**Supplementary Information:**

The online version contains supplementary material available at 10.1007/s11356-023-30253-w.

## Introduction

Resource islands (RIs) or fertility patches are vegetation-dominated patches in arid and semi-arid regions, typically characterized by the presence of a nurse tree or shrub (Mudrak et al. 2014). These RIs play a vital role in enhancing soil moisture, nutrient availability, vegetation productivity, and soil microorganism diversity. While microorganisms in these regions are exposed to extreme conditions, their exposure to heavy metals (HMs) is relatively limited, except in areas affected by anthropogenic sources (Shi et al. 2023) as noted by Wang et al. (2022). However, it is important to acknowledge that the diversity and activity of microorganisms in arid zones can be influenced by the presence of HMs (Zeng et al. 2020). For instance, dominant phyla found in RIs soils, such as Bacteroidetes and Saccharibacteria, have shown resistance to HMs including Cr, Zn, Pb, Cu, and Ni (Khan et al. 2015). Hence, microorganisms derived from RIs hold potential as bioremediators for contaminated soils.

Soil microorganisms possess the inherent capability to resist and tolerate HMs. Resistance refers to their ability to thrive in the presence of constant inhibitory concentrations of a substance, while tolerance refers to their capacity to remain dormant and survive in environments containing the substance without significant growth (Muñoz-García et al. 2022). Some HMs play essential roles in metabolic reactions, while others pose risks to the environment, human health, and microbial diversity (Qi et al. 2022). The principal mechanisms underlying tolerance and resistance include the formation of stable complexes with biosurfactants and active excretion of accumulated metals outside the cell through efflux transporters (Seneviratne et al. 2017). Another mechanism involves gene regulation facilitated by highly modified genetic systems, utilizing proteins that aid in metal detoxification (Verma and Kuila 2019). Within this context, metabolic adjustments play a pivotal role as bacteria adapt their metabolism to mitigate HM toxicity (Han et al. 2021).

Bacteria that exhibit resistance and tolerance to heavy metals (HMs) play a vital role in promoting plant survival and establishment, particularly in challenging conditions like drought and metal stress (Ma et al. 2016). For instance, the plant growth-promoting bacterium (PGPR) *Brevibacillus* offers protection to plants when exposed to Cd and Zn stress (Azcón et al. 2013). Similarly, bacteria such as *Pantoea stewartii*, *Microbacterium arborescens*, and *Enterobacter* confer resistance to Cr and drought conditions in mesquite tree species (*Prosopis juliflora*) (Khan et al. 2015). In arid soil from northeastern Algeria, *Rhizobium* promotes resistance to As, Zn, and Cu in plants like *Genista microcephala* and *Argyrolobium uniflorum* (Dekak et al. 2018). Genes responsible for resistance to Zn, Cr, Ni, and Hg, such as *czc*, *chr*, *ncc*, and *mer*, have been identified through metagenomics in Brazilian soil with no reports of HM contamination (Gallo et al. 2019). Additionally, genes like *copA* confer resistance to Cu, *czcA* to Co, Zn, and Cd, while other genes such as *cznt*, *pbrB*, *pbrA*, *pbrT*, *pbrR*, *troB*, and *nmtR* are associated with lead resistance and/or tolerance. Moreover, the *zraS* gene has been linked to zinc resistance in arid soils naturally contaminated with HMs (Liu et al. 2022a).

Although the role of microorganisms in RIs in terms of resistance and tolerance towards HMs is relatively unexplored (Bashan et al. 2008), previous research has demonstrated the ability of mycorrhizal fungi present in RIs to resist HMs (Gonzalez-Chavez et al. 2009). In light of this, our study hypothesized that microorganisms in RIs may possess adaptation mechanisms that enable them to withstand stress conditions such as pH, salinity, and temperature, thereby naturally conferring resistance and tolerance to HMs that occur at low concentrations in semiarid soils. Therefore, the objective of this study was to evaluate the resistance and tolerance capacity of soil bacteria in RIs within the semiarid zone of the Colombian Caribbean region towards HMs. To achieve this, we conducted high-throughput sequencing of soil microorganisms at three nurse tree sites during both the dry and wet seasons, encompassing both vegetated and unvegetated soils. Subsequently, we established correlations between the most abundant genes of interest and physicochemical parameters, as well as the taxonomic and functional diversity of the soil. Additionally, bacteria were isolated and exposed to varying concentrations of HMs to determine their tolerance mechanisms through genomics. This study contributes to our understanding of the behavior and mechanisms employed by microorganisms in RIs to thrive in the presence of HMs. Furthermore, it provides valuable insights for the development of soil remediation strategies in semiarid regions and the formulation of conservation policies.

## Materials and methods

### Sampling

Surface soil samples were collected from beneath the canopy of three nurse tree species: *Prosopis juliflora* (Tru), *Pithecellobium dulce* (Tor), and *Haematoxylum brasiletto* (Bra) which were the dominant nurse trees in the study area. The samples were collected at a depth of less than 2 cm, with the aim of capturing a superficial black-colored organic layer distributed in a patch-like manner between the nurse tree and the canopy's edge. Each integral sample was composed of subsamples of individual trees, resulting in a total of nine samples (three islands sampled for each tree species). For subsequent analysis, DNA extraction was performed on subsamples weighing up to 50 g. Additionally, 1 kg of soil was collected for physicochemical analysis. The analysis considered two conditions: vegetation-free soil (C) and soil with vegetation (V), with vegetation-free soil serving as the control. Sampling was conducted at the same locations during both the dry season (D) and wet season (W) to investigate the response of microorganisms under different environmental conditions.

### Soil physicochemical analysis

A total of 12 samples were collected, consisting of composite samples from each nurse tree and control group. These samples were analyzed to assess various soil physicochemical properties. The pH of the soil was measured using a pH meter, while electrical conductivity (EC) was measured through the 1:5 soil-water extraction technique and quantified using potentiometry. Organic matter (OM) was measured using visible spectrophotometry. Exchangeable bases in the soil (Ca, Mg, K, and Na) were measured using the 1M ammonium acetate extraction technique at pH 7.0 and quantified using atomic absorption spectrophotometry. Available phosphorus was measured using the Bray II extraction technique and quantified using UV-VIS spectrophotometry. Microelements in the soil (Fe, Cu, Mn, Zn) were measured using the Olsen solution extraction technique and quantified using atomic absorption spectrophotometry. Available sulfur was measured using the VIS-Turbidimeter spectrophotometry technique, while available boron was measured using the VIS-Extraction spectrophotometry technique. The effective cation exchange capacity (CEC) was determined through calculations, and soil texture was measured using the Bouyoucos technique.

### Metagenomic analysis of HMs resistant and tolerant microorganisms

DNA extraction from each sample was performed using the Stool DNA kit (OmegaBio-tek, Norcross, GA), and the concentration of total DNA was measured using a Qubit 2.0 fluorometer. To assess DNA quality, a 1% agarose gel electrophoresis test was conducted. The metagenome sequencing was carried out on the DNBseq platform by BGI NGS. The raw sequence quality was assessed through FASTQC. Readings with a PHRED quality score of <30 were selected, and those below 80 bp in size were excluded using the 'iu-filter-quality-minoche' program (Eren et al. 2013). For functional classification, alignments were executed against NCBI's Non-Redundant Protein Database using DIAMOND v0.9.29.130 (Buchfink et al. 2015). The alignments were mapped against megan-map-Jan2021.db, which includes the KEGG database. The results were visualized and compared in MEGAN6 (Huson et al. 2007). The functional analysis was performed considering the reaction module of the Kyoto encyclopedia of genes and genomes - KEGG (Kanehisa and Goto 2000).

### The metagenome-assembled genomes (MAGs)

The metagenomic reads were assembled using MEGAHIT v1.1.3 (Li et al. 2016) with k-mer size from 99 to 255, removing contigs smaller than 1000bp. Genome binning was performed using Metabat2 v2.12.1 (Kang et al. 2019), MaxBin2 (Wu et al. 2016) v2.2.5 and CONCOCT (Alneberg et al. 2013). The metagenome-assembled genomes (MAGs) obtained using these approaches were consolidated with DAStool (Sieber et al. 2018). The completeness and possible contamination of each MAG was verified using CheckM v1.0.11 (Parks et al. 2015). The taxonomic classification was performed using GTDB-tk (Chaumeil et al. 2020). For each MAG, we carried on the functional annotation using Prokka v.1.14.5 (Seemann 2014). The KOs associated with HMs resistance and tolerance detected in the metagenome were searched in all MAGs to assign them a taxonomic category. The heatmaps were generated using the Tb Tools software (Chen et al. 2020).

The list of genes associated with HMs tolerance and resistance mechanisms was constructed using KEGG. To differentiate between tolerance and resistance genes, those linked to efflux mechanisms, including membrane transporter proteins and efflux pumps, were categorized as tolerant (Dunlop et al. 2011). In the case of resistance genes, consideration was given to those associated with enzymes that metabolize the metal to synthesize a product, P-type transporters indicating detoxification systems (Nies 1999), oxidoreduction activities, or those previously identified as resistance genes within the KEGG database. By conducting a bibliographic review of research articles and utilizing the BacMet database (http://bacmet.biomedicine.gu.se/), we verified the resistance annotation of the genes identified in KEGG. All genes detected in the metagenome associated with tolerance and resistance to HMs were queried against the UNIPROT database (https://www.uniprot.org/) to ascertain their molecular functions. These genes were then linked to their respective KO terms. To analyze the relationship between physicochemical variables and genes, Pearson correlation and principal component analysis (PCA) were performed using RStudio software.

### Characterization of HMs resistant bacterial isolates

To identify bacteria resistant to HMs, we conducted two continuous enrichments in PAF liquid medium at 30°C and 150 rpm for 24 h. Soil samples were collected from three RIs and bare soil. Bacteria from the primary culture were then replicated in PAF medium and incubated at 50°C for 24 h. To preserve them for future use, the bacterial strains were stored in 25% glycerol at -80° C. Among the bacterial strains, C3-3 and T106, which exhibited robust growth at 50° C, were selected for evaluating their tolerance to HMs. The tolerance of these bacteria to HMs was assessed in triplicate using Luria Bertani (LB) broth supplemented with four concentrations of Cd, Co, Mn, and Ni (0.5 mM, 1.0 mM, 5.0 mM, and 10.0 mM) at 25°C for 2 d, following the method described by Andrews (2001). Growth was measured by optical density at 600 nm using spectrophotometry in 96-well plates at 24 and 48 hours of incubation. For further analysis, the HMs-resistant bacteria underwent sequencing to determine their taxonomic identity and the mechanisms underlying their resistance and tolerance to HMs. DNA extraction was performed using the DNeasy PowerSoil kit from Qiagen, and sequencing was conducted on the Illumina NovaSeq6000 platform. The primary reads were analyzed using FastQC v0.11.9, and genome assembly was performed using Unicycler v0.4.8. Annotation of the genome was accomplished using RASTtk (Brettin et al. 2015) through the BV-BRC v3.28.21 platform (Davis et al. 2020). Genome contamination was evaluated based on the 16S rRNA gene sequence using EzBioCloud (Yoon et al. 2017). The functional assignment of the proteins was performed using the KEGG KOs (KEGG Orthology) database (Kanehisa and Goto 2000), and proteins associated with resistance and tolerance to HMs were identified as previously described.

## Results

### Abundances of HM resistance genes

**Fig. 1:**
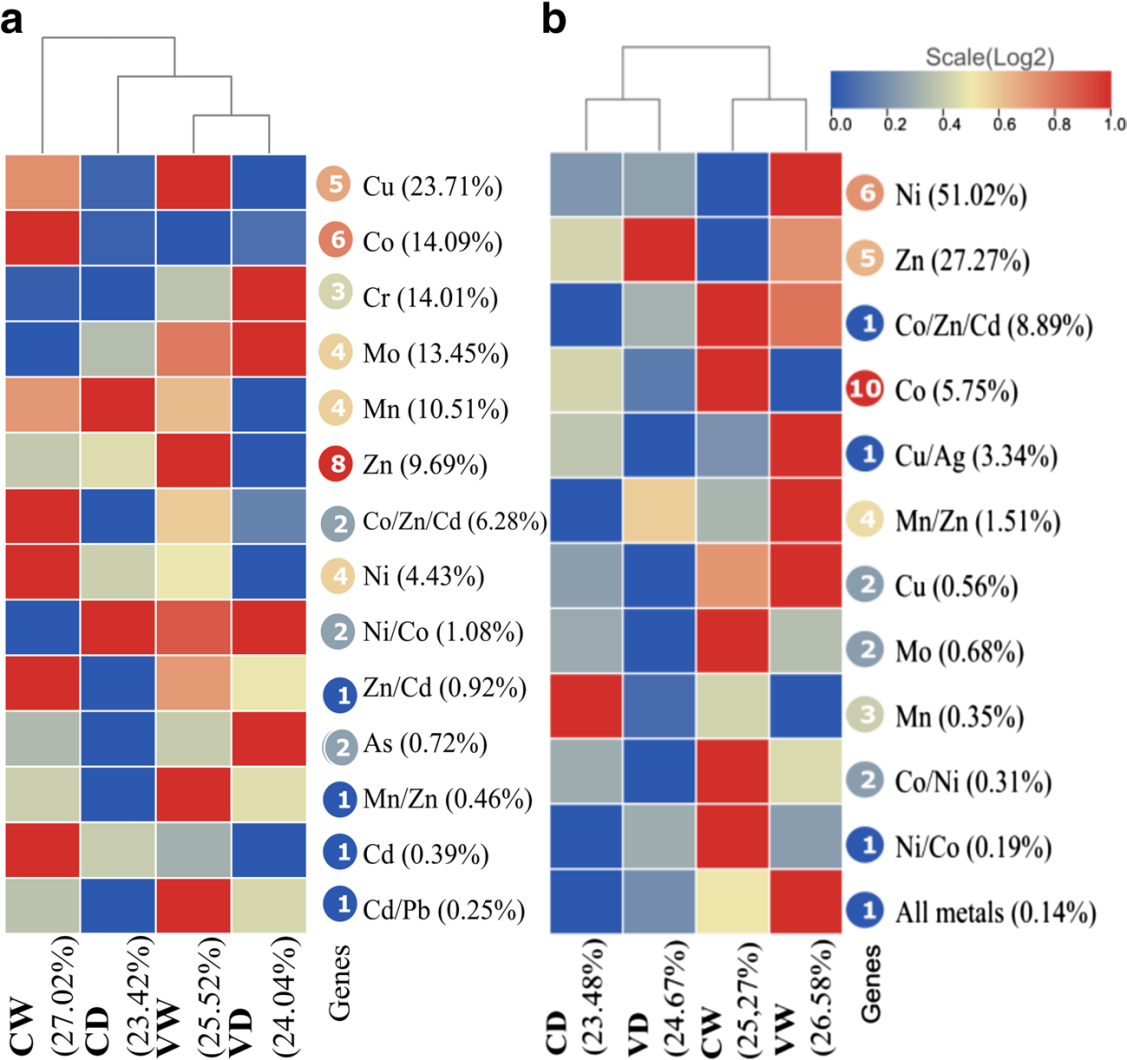
Heatmap of abundances of resistance-associated genes **(a)** and tolerance-associated genes **(b)** to HMs under four conditions, dry season (D) and wet season (W), with vegetation (V) and bare soil (C)

The metals Cu, Co, Cr, Mo, and Mn showed the highest abundance of resistance genes, particularly during the wet season with vegetation (Fig. 1a). The genes copA, ctpA, and ATP7 had the greatest abundances (20.63%), and significant differences were observed among the different conditions, these genes were associated with resistance to Cu. The genes yagR and chrA ranked second and third in abundance, respectively, and were associated with Mo and Cr. Additionally, the ARSB gene demonstrated both resistance and tolerance and is linked to arsenic metabolism. Notably, the most significant differences in gene abundance were observed in dry and vegetation-free conditions. The VW and DC conditions had the highest abundances of resistance genes (Tables 1 and 2). Among the resistance genes, copper-binding proteins displayed the highest prevalence (20.53%), followed by oxidoreductase activity with the second highest abundance (20.52%), specifically associated with Cr, Mn, and Co. Furthermore, the chromate transmembrane transporter exhibited the third highest abundance (8.37%). Most of these molecular functions were influenced by seasonality, with dry soil (D) being the most favorable condition for their overall development. Bare soil (C) followed as the next suitable condition. The optimal condition for the thriving of these molecular functions was found to be dry soil without vegetation (DC) (Table S1).

**Table 1:** Abundance of HM resistance genes in RIs during dry (D) and wet (W) seasons under vegetation presence (V) or absence (C)

Gen (Metal)	Abundances (%)	Condicions				P-value			
		VW	CW	DV	DC	1	2	3	4
*copA, ctpA, ATP7* (Cu)	20.63	9599	9437	8691	9039	*	**	*	+
*yagR* (Zn)	11.28	4980	3008	5976	4078	+	+++	***	*
*chrA* (Cr)	8.41	3549	3921	3903	3538		++		
*mntH* (Mn)	7.19	3091	2936	3314	3680		+++		
*cobN* (Co)	6.49	2332	5539	2467	3117	***		+++	
*chrR*, *NQR* (Cr)	4.84	2354	1699	2145	2017		**	+++	
*czcD*, *zitB* (Co/Zn/Cd)	4.02	1865	1747	1759	1679		***		
*cobT* (Co)	4.01	1634	895	2216	1748		***	***	
*TC, ZIP* (Zn)	3.58	1516	1910	1531	1867	+	**		
*yydH* (Zn)	2.68	1325	991	1123	1176		+		+
*arsB* (As)	0.51	211	181	273	227				
Final totals		42343	44132	41063	40182	(*) 5	9	7	2
						(+) 5	5	4	4

*P* Value: 1: VC (+: V>C); 2: WD (+: D>W); 3: WC vs WV (+: WC > WV); 4: DV vs DC (+: DC > DV); *:It indicates the opposite of what was previously stated

Significant differences: ***/+++: *p*<0.001; **/++: *p*<0.01; */+: *p*<0.05

Total resistant genes: 43. The gen arsB presented resistente y tolerante to HMs

Total abundance sum of the metagenome: 3988563

**Table 2:** Abundance of genes for HM tolerance in RIs during dry (D) and wet (W) seasons under vegetation presence (V) or absence (C)

Gen (Metal)	Abundances (%)	Condicions				P-value			
		VW	CW	DV	DC	1	2	3	4
*pqqL* (Zn)	20.78	17377	12631	19874	15733	+	+++	*	
*ABC,PE,S* (Ni)	17.13	15961	14002	13317	12864	+++	*		
*ABC,PE,P* (Ni)	10.01	8663	7959	8384	8316				
*czcA, cusA, cnrA* (Co/Zn/Cd)	8.89	8767	9943	6204	5034	*			
*ABC,PE,A1* (Ni)	8.81	7226	7305	7661	7370		+++		
*ABC,PE,P1* (Ni)	8.68	7609	7374	6953	7311				
*ABC,PE,A* (Ni)	6.04	4926	4265	5359	5648		***		
*yahK* (Zn)	4.6	3862	2351	4612	3148	++	+++	**	**
*cusA, silA* (Cu/Ag)	3.34	3426	2544	2361	2715				
*cobS* (Co)	1.89	1298	828	2095	1763			*	
Final totals		85897	78515	82202	75989	(*) 11	7	7	2
						(+) 4	6	6	4

*P* value: 1: VC (+: V>C); 2: WD (+: D>W); 3: WC vs WV (+: WC > WV); 4: DV vs DC (+: DC > DV); *:It indicates the opposite of what was previously stated

Significant differences: ***/+++: *p*<0.001; **/++: *p*<0.01; */+: *p*<0.05

Total tolerant genes: 38

Total abundance sum of the metagenome: 3988563

Numbers in a circle correspond to the number of genes associated with every metal. Besides, the number in parenthesis close to the metals mean the abundance’s percentage in genes associated to each metal.

### Abundances of genes and metabolism of HMs tolerance

The metals exhibiting the highest tolerance abundances were Ni, Zn, Co/Zn, Cd, Co, and Cu/Ag (Fig. 1B). The most abundant tolerant genes were detected under humid conditions (CW and VW). Mercury showed the lowest tolerance capacity (0.14%). The highest tolerances were observed for Zn and Ni (Table 2), with the pqqL gene (Zn) being the most abundant at 20.8%. ABC genes were associated with Ni metabolism and showed variations in relation to seasonality (W-D), while czcA, cusA, and cnrA genes were only influenced solely by the presence or absence of vegetation (V-C). Although no significant differences were observed in the presence or absence of vegetation, the most prevalent conditions were CW and VD, corresponding to p-values 3 and 4. The molecular function hydrolase/protease (Zi) displayed the highest abundance, followed by the peptide transmembrane transporter (Ni) and hydrolase (Ni) (Table S3). Activities related to cation transmembrane transporter did not show significant differences, unlike hydrolase/protease and peptide transmembrane transporter. Oxidoreductase and methyltransferase functions exhibited differences in 4 and 3 out of the 4 p-values, respectively. Overall, no differences were found between seasonality (p-value 1), and vegetated soil was the most abundant (*p*-value 2). The two significant conditions were WC and DV, similar to the genes with tolerance capacity (Table 2).

### Correlation of tolerance and resistance genes with physicochemical variables

**Fig. 2:**
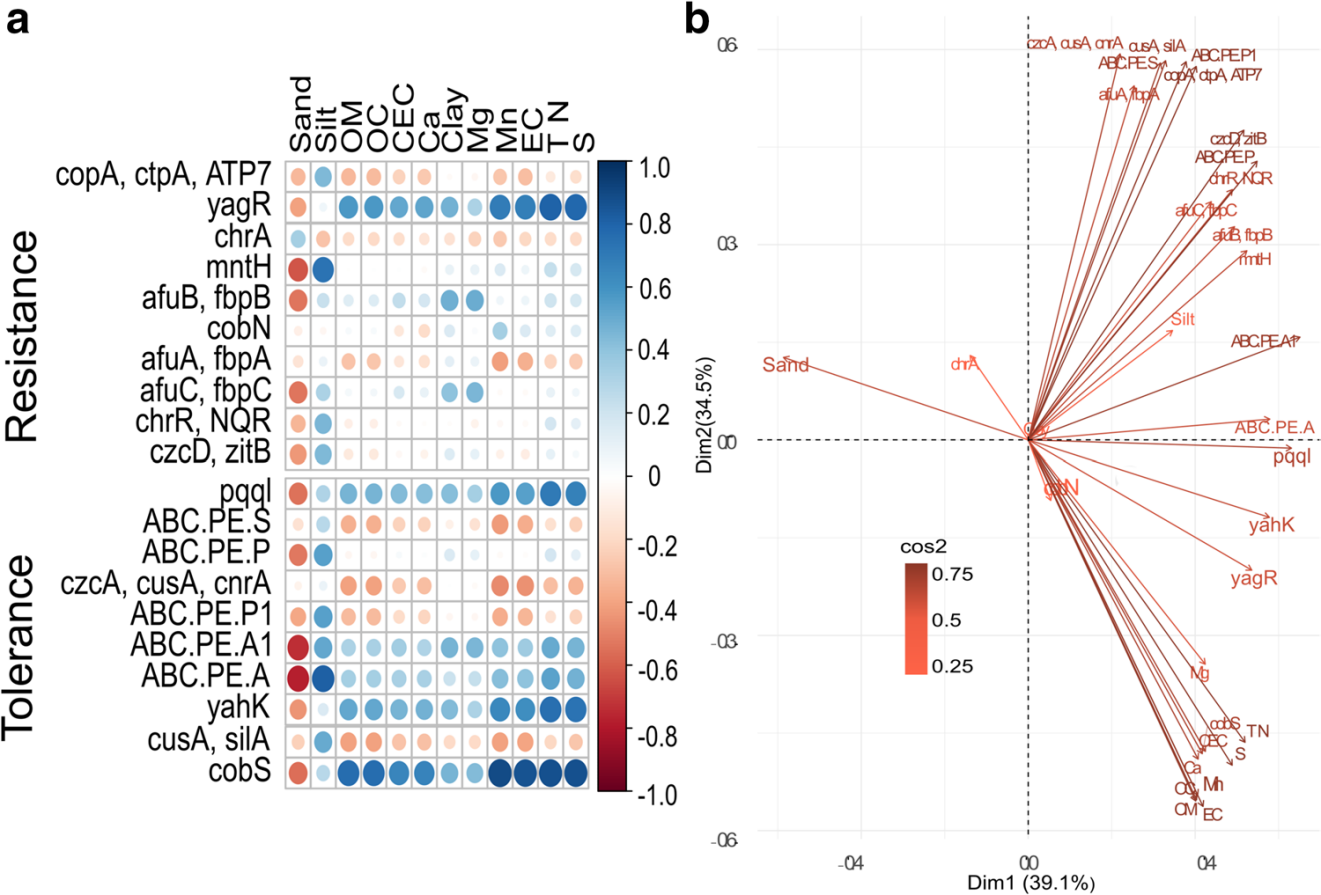
Pearson correlation of physicochemical variables with the most abundant resistance and tolerance genes (**a**) and principal component analysis (**b**). Pearson correlation between physicochemical parameters with the six most abundant genes associated with tolerance and the six most abundant resistance genes in the resource islands. Electrical conductivity (EC), organic carbon (OC), total carbon (TC), total nitrogen (TN), calcium (Ca), potassium (K), magnesium (Mg), sodium (Na), effective cation exchange capacity (ECEC), phosphorus (P), sulfur (S), copper (Cu), iron (Fe), manganese (Mn), zinc (Zn), and boron (B) with their respective correlation coefficient “r” in the right bar

The majority of resistance genes displayed weak positive correlations with physicochemical parameters, except for the *yagR* (Mo) resistance gene, which showed stronger correlations with nearly all variables such as clay, TN, EC, and Mn, among others. The only exception was the sand parameter (Fig. 2a). The tolerant gene *cobS* also showed stronger correlations than the resistance genes except for the sand parameter. This sand parameter had negative correlation with all genes that were observed; nonetheless, in for *chr*A (Cr), it still showed a positive correlation. On the other hand, clay exhibited the highest positive correlation with both resistance and tolerance genes. Overall, tolerant genes showed better correlations with physicochemical parameters compared to resistance genes.

### Taxonomic assignment of tolerance and resistance genes detected in RIs

**Fig. 3:**
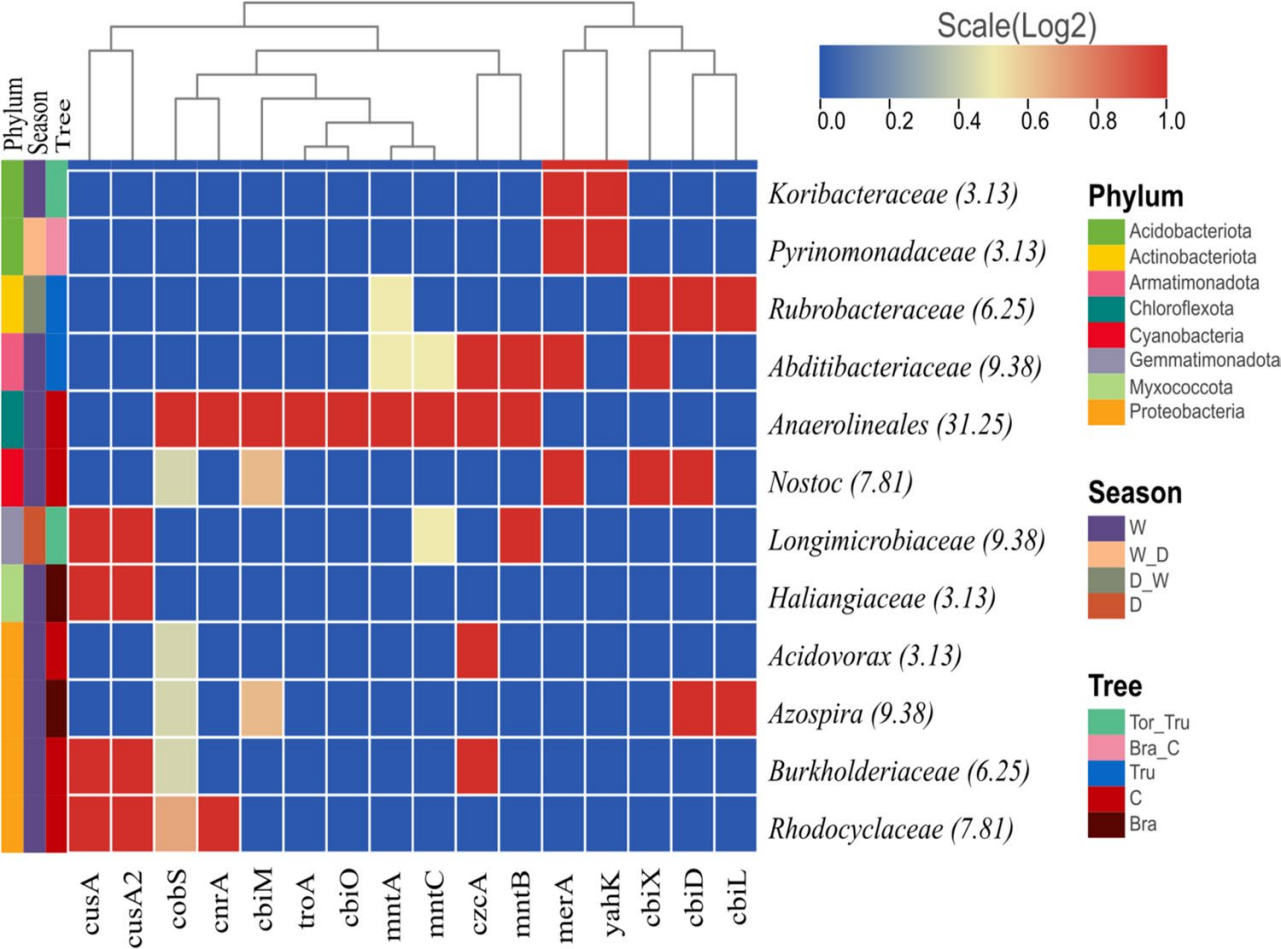
Heatmap of the correlation between 16 tolerant genes and taxonomic assignment. The percentage of gene presence is shown in each taxonomic category

**Fig. 4:**
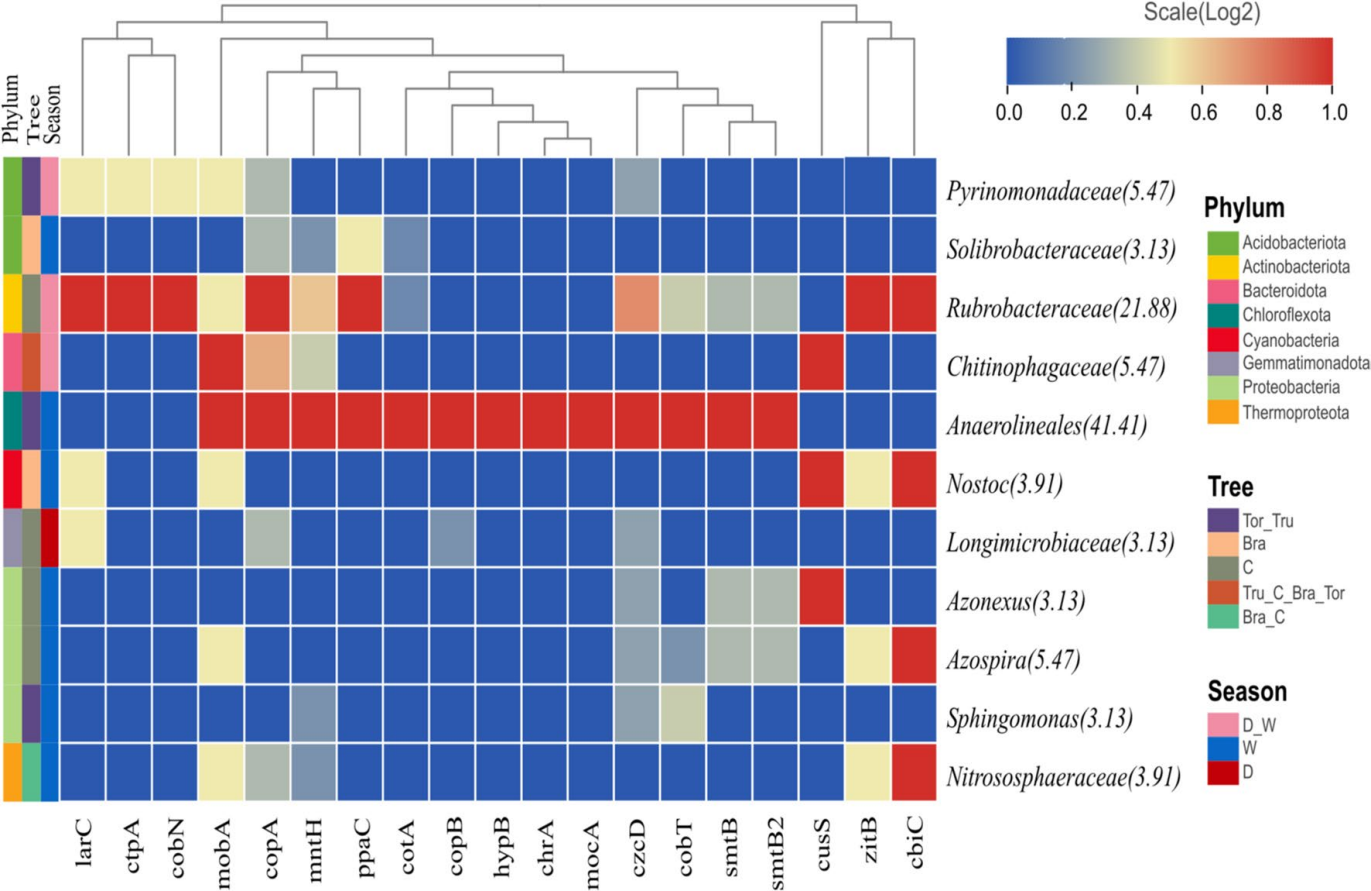
Heatmap of the correlation between 19 resistant genes and taxonomic assignment . The percentage of gene presence is shown in each taxonomic category

Seventy-five MAGs were reconstructed and categorized into 34 taxonomic groups. The taxonomic assignment of tolerance genes was low (Fig. 3). Anaerolineales showed the highest abundance of tolerance genes (31.25%), while the families *Abditibacteriaceae, Longimicrobiaceae*, and *Azospira* exhibited an equal abundance of 9.38% each. The *cobS* gene was detected in multiple bacterial categories, but with lower abundance levels. Notably, the wet season displayed higher abundance levels across all categories (Fig. 3). Regarding the assignment of resistance genes, the order Anaerolineales (phylum Chloroflexi) had the highest assignment (41.41%), followed by the order Rubrobacteraceae (phylum Actinobacteria) with 21.88% (Fig. 4). Several taxonomic categories associated with the phylum Proteobacteria were also identified in third place. The genes *mobA* and *copA* were found in multiple taxonomic orders, while *ctpA* and *cobN* showed an association within the same cluster and were present in two similar taxonomic orders with similar abundance levels. Similar to the findings for tolerance genes, the wet season exhibited higher abundance levels for resistance genes (Fig. 3 and 4).

### Culturable bacteria

**Fig. 5:**
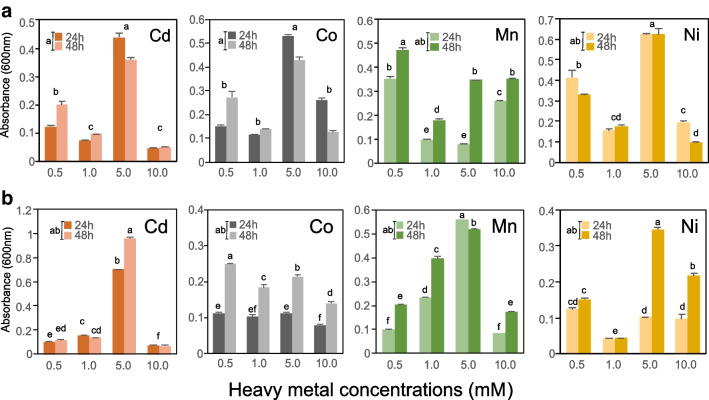
Growth of *C. gottheilii* T106 (**a**) and *B. velezensis* C3-3 (**b**) at 0.5, 1.0, 5.0, and 10.0 mM of Cd, Co, Mn, and Ni at 24 and 48 h. The bars represent the mean with their respective standard error. Different letters indicate significant differences through Tukey's mean comparison, with a *p*-value ≤ 0.05

The isolates C3-3 and T106 were identified as *B. velezensis* and *C. gottheilii*, respectively, and their genomes were assembled into 41 and 44 contigs, respectively. The genome of *C. gottheilii* T106 contained 70 tRNA genes, two rRNA genes, and 5440 CDS, while *B. velezensis* C3-3 had 78 tRNA genes, two rRNA genes, and 4051 CDS. Both bacteria possessed genes associated with the transport and metabolism of HMs. T106 had a higher number of cobalt-related genes (8 genes), whereas C3-3 had more genes related to nickel, iron (57 genes), and copper (49 genes). Both bacteria demonstrated growth at various concentrations of HMs (Cd, Co, Mn, and Ni). T106 exhibited better growth at 0.5 and 5.0 mM concentrations in both time ranges, while C3-3 thrived better at 5.0 mM concentrations of Cd and Mn at 24 and 48 hours (Fig. 5).

## Discussion

In the study area, trees of *H. brasiletto* and *P. dulce* exhibit sclerophyllous strategies, characterized by compact and thin leaves, high leaf dry matter content, and elevated photochemical performance. These traits enable them to endure water scarcity and high temperatures in semi-arid environments (Toro-Tobón et al. 2022). Dominant leguminous plants in the region often establish beneficial relationships with nitrogen-fixing rhizobacteria, enhancing their adaptation to oligotrophic environments. These plants contribute shade and organic matter to the soil, creating favorable conditions for microorganisms that actively engage in nutrient cycling (Bashan et al. 2008) and potential metabolic activities, including resistance and tolerance to heavy metals.

### Genes related to HM resistance

Anaerolineales, belonging to the Phylum Chloroflexi, exhibited the highest levels of resistance (31.25%) and tolerance (41.41%; Fig. 3 and 4). This bacterial group is known for its versatility and is commonly found in ecosystems contaminated with multiple pollutants, including HMs (Yu et al. 2020). They are involved in the stabilization of metal ions through sulfate reduction, contributing to metal detoxification (Zhang and Shan 2021). Among the resistance genes, *copA*, *ctpA*, and *ATP7* (20.63%), *yagR* (11.28%), and *chrA* (8.41%) displayed the highest abundances (Table 1). These genes are known to be associated with microbial resistance to copper (Cu), and their presence correlates with the abundance of Cu-binding proteins (Table 2). The *copA*, *ctpA*, and *ATP7* genes encode copper-transporting P-type ATPases, which act as primary barriers to prevent Cu entry into the cytosol and play a significant role in cellular detoxification of Cu (Li et al. 2015). Consistent with previous studies, Cu resistance-related genes were found to be among the most abundant, indicating the importance of Cu as one of the primary metals to which genes develop resistance. Zn is another metal that has been reported to be frequently associated with high gene abundances for resistance (Liu et al. 2022a). P-type ATPases play a significant role in cellular adaptation and response to environmental stress across diverse organisms. These proteins are crucial in managing variable and fluctuating conditions in bacteria, serving as key components in their environmental response mechanisms (Muñoz-García et al. 2022).

The *yagR* gene, associated with Mo resistance, exhibited the second highest abundance among the genes analyzed (Table 1). This gene encodes the molybdenum-binding subunit of the xanthine dehydrogenase protein known as YagR (Kanehisa and Goto 2000). Mo was identified as the fourth most important metal associated with resistance genes (Fig. 1**a**). The xanthine oxidase family, which contains the molybdenum cofactor (Mo-Co), is a diverse group of enzymes. Xanthine dehydrog enase, an oxidoreductase, is one of the enzymes in this family (Kanehisa and Goto 2000). Bacteria adapt to thermal and arid conditions by upregulating stress-responsive genes, such as oxidoreductases (Ren et al. 2018). In this study, the *yagR* gene, along with *chrR/NQR*, *cotA*, and *corC* genes, was found to be associated with metal resistance to Mo, Cr, Mn, and Co (20.52%). These enzymes play a crucial role in breaking the bonds of toxic elements and utilizing the energy generated by biochemical reactions. This process enables the oxidation of harmful contaminants into harmless compounds by transferring electrons to other chemical compounds (Jacob et al. 2018). The *chrA* gene, which exhibited the third highest abundance (Table 1), encodes a chromate reductase responsible for conferring resistance to chromium (Cr), the third most abundant metal identified in this study (Fig. 1a). This gene is considered a marker for the selection of Cr (VI)-resistant bacteria and functions as a chemiosmotic pump, using proton motive force to extrude chromate from the cell cytoplasm (Ontañon et al. 2018). Several members of the CHR superfamily, including chromate transporters, play a role in conferring Cr resistance by functioning as transmembrane pumps (Aguilar-Barajas et al. 2012). These findings align with the top three molecular functions associated with metal resistance identified in this study (Table S2).

### Abundance in tolerance and metabolism of HMs

The tolerance genes exhibiting the highest abundance were the ABC transporters (51.67%), followed by *pqqL* (20.78%), and *czc*, *cusA*, and *cnrA* at 8.89% (Table 3). ABC transporters are specifically associated with Zn, which is the metal displaying the largest number of tolerance genes (Fig. 1b). These transporters often consist of a substrate-binding protein (SBP) subunit that determines their specificity and high affinity for ABC uptake systems in bacteria (Maqbool et al. 2015). These proteins in bacteria help enhance adaptation to limiting conditions by facilitating the absorption of essential nutrients, such as carbohydrates and phospholipid precursors (Chandravanshi et al. 2019). Furthermore, the most abundant molecular function was peptide transmembrane transporters (18.69%; Table S3). Peptides play a role in removing HMs from the environment by forming complexes with proteins and metal ions. For instance, metallothioneins, cysteine-rich polypeptides, exhibit binding affinity towards HMs such as Cd, Zn, Hg, Cu, and Ag (Ullah et al. 2015).

**Table 3:** Mechanisms involved in the resistance and tolerance to heavy metals of *C. gottheilii* T106 and *B. velezensis* C3-3 isolates

Metals	Mechanisms	# of genes (%)	
		T106	C3-3
Cu	Homeostasis protein (1)^T^, transcripcional regulator (1)^C^, cytochrome (15)^T, C^, and transporter protein (1)^T^ (2)^C^.	17 (5)	18 (7)
Others	Lactate dehydrogenase (1)^T^, nitrate reductase (1)^T, C^, succinate dehydrogenase (1)^T, C^, cytochrome oxidase (1)^T,C^, chromate transport protein (1)^T, C^, thioredoxin reductase (1)^T, C^ , and dehydrogenase (1)^C^.	6 (1)	6 (1)
Zn	Transcriptional regulator (2)^TC^, dehydrogenase (1)^C^ , and transporter protein (4)^T^ (3)^C^.	6 (2)	6 (2)
Co	Methyltransferase, hydrolase, chelatase, reductase, and methylmutase.	8 (3)	0 (0)
Ni	Transporter protein.	4 (1)	4 (1)
Mn/Zn/Fe	Transporter protein.	4 (2)	4 (2)
Mo	Cofactor (2)^T, C^, and nitrate reductase (1)^C^.	2 (1)	3 (1)
Co/Ni	Transporter protein.	4 (1)	0 (0)
Zn	Chromate reductase (1)^C^, chromate transporter protein (1)^T, C^.	1 (0)	2 (0)
As	Arsenate reductase	1(0)	1 (0)
Cd/Zn	ATPase	1 (0)	1 (0)
Cd/Co/Zn	Proteína de sistema de eflujo	1 (0)	1 (0)
Cd	Cytochrome	0 (0)	1 (0)
Fe	Transcripcional regulator	1 (0)	0 (0)
Mn	Transporter protein	0 (0)	1 (0)
Pb/Cd/Zn/Bi	Transcripcional regulator	1 (0)	0 (0)

The numbers in parentheses in the process column correspond to the number of genes in each mechanism, and the superscripts T: T106 and C: C3-3

The *pqqL* gene ranked as the second most abundant, encoding a putative metallopeptidase periplasmic protease that is expressed under iron-limiting conditions and confers tolerance to Zn (Grinter et al. 2019). This activity was associated with the hydrolase-protease function (Table 2). These enzymes are crucial in the metabolism of HMs as they withstand its destabilizing effect (Kaplia 2016). Conversely, the *czca*, *cusA*, and *cnrA* genes displayed the third highest abundance (Table 3). Collectively, these genes are linked to tolerance against Co, Zn, and Cd. This tolerance mechanism involves an efflux system facilitated by the RND (resistance-nodulation-cell division) protein complex, which transports metals from the cytoplasm and expels them into the extracellular medium (Zieliński et al. 2021). Mechanisms of the outer membrane protein efflux system, including the proteins encoded by *czca*, *cusA*, and *cnrA* (Kanehisa and Goto 2000), along with cation transmembrane transport, were among the top five molecular functions associated with tolerance (Table S3). The efflux mechanism in bacteria promotes their survival in arid environments by enabling the expulsion of harmful substances and maintaining cellular homeostasis. This adaptation is crucial for their endurance in harsh climatic conditions characterized by high temperatures and limited water availability (Soares et al. 2012).

Bacteria generally exhibited higher resistance levels rather than tolerance to HMs, suggesting that these microbial communities harness their adaptive mechanisms more effectively to withstand the presence of these elements. For instance, adaptation to salt stress involves mechanisms such as efflux pumps, siderophore secretion, membrane proteins, and metallopeptidase activity (Liu et al. 2022b). It is important to note that the abundances of metals associated with resistance differ from those associated with tolerance. Metals such as As, Cd, Pb, Cr, Ni, Zn, Al, and Mn have been identified as the most detrimental in terms of environmental pollution (Jacob et al. 2018) and showed higher abundances in resistance, while Zn and Ni were predominantly present in genes associated with tolerance (Fig. 1b). Bacteria can develop tolerance to these elements, except for Cr, which was observed among the top three metals associated with resistance (Fig. 1a). The efflux system consists of two chromate efflux proteins encoded by the *chr*A gene. On the other hand, the reduction of Cr(VI) to Cr(III) is carried out by extracellular soluble reductases dependent on NAD(P)H. Cr(III) is eliminated by reacting with functional groups present in the cell (Ahemad 2014). In bacteria, NAD(P)H-dependent soluble reductases play a vital role in their survival within arid environments. They facilitate the transfer of reducing equivalents and maintain redox balance, thus proving essential for bacterial adaptation to such conditions (Phillips 2007).

The *arsB* gene is unique in its capability to confer both resistance and tolerance. In general, arsB encodes the membrane protein arsenic pump, which functions through the efflux system, expelling As^3+^ (arsenite) out of the cell (Sher and Rehman 2019). The molecular function of the protein is associated with the active transmembrane transporter of arsenite (Table S2). The product of the *arsA* gene is an arsenite-stimulated ATPase that, when associated with arsB, forms a complex that functions as an anion-translocating ATPase (Pillai et al. 2014). Additionally, higher taxonomic abundances were observed during the wet season (Fig. 3 and 4), coinciding with the abundances of tolerant and resistant genes and molecular functions (Tables 1 and 2). This aligns with several reports from various soils indicating that soil moisture increases fertility and, consequently, the microbial diversity that inhabits it (Reyes and Cafaro 2015). Acidobacteria and Actinobacteria exhibited the highest abundances for the genes of interest (Fig. 3 and 4). These bacteria have been detected in soils contaminated with HMs (El Baz et al. 2015). A similar case is observed in Proteobacteria, as several taxonomic categories associated with it were identified in the genes of interest. This phylum, along with Actinobacteria, has been found to be highly abundant in HM-contaminated gold tailings (Liu et al. 2022c).

During the wet season, resistance and tolerance genes exhibited the highest abundances, particularly in the presence of vegetation (Table 1 and S2). Tan et al. (2023) demonstrated that bacterial enzymes in rewetted soils increase their tolerance to cadmium and other HMs compared to naturally dry soils. It is important to note that optimal moisture levels are necessary for effective detoxification of toxic chemical elements (Borowik and Wyszkowska 2016). Conversely, negative relationships were observed between tolerance and resistance genes with sand, while positive relationships were found with clay and silt. This pattern can be attributed to the capacity of fine-grained soils to form bonds (Matos et al. 2017). Clays have a tendency to retain HMs, which may exert selective pressure favoring the presence of tolerance and resistance genes. This retention is achieved through cation exchange in the intermediate layers resulting from interactions between ions (metals) and the permanent negative charge (Olaniran et al. 2013). For example, Li et al. (2017) showed that clay efficiently retains metals such as Zn, Cd, Pb, and Cr, whereas metal retention in sand is less stable, offering less physical protection. Consequently, sand exerts less selective pressure on tolerance and resistance genes. Rajmohan et al. (2014) reported a negative correlation between sand and EC, Fe, Zn, and Ni parameters, while clay and silt showed a positive correlation with the same parameters.

However, in this study, sand exhibited a slightly positive correlation with the chrA gene associated with chromium resistance, despite the chrR gene, which is also associated with Cr resistance, not showing a positive correlation. This discrepancy could be attributed to the higher abundances of the *chrA* gene (Table 1) and the greater effectiveness of the chromate efflux protein (ChrA) in MP detoxification compared to *chrR*. This could be because the chromate reductase '*chrR*' generates a large amount of reactive oxygen species (ROS), resulting in minimal reduction of Cr (Thatoi et al. 2014). Similarly, negative correlations were observed between tolerance and resistance genes and positive cations such as Ca and Mg, which compete with each other. Consequently, higher abundance of these cations could indicate lower concentrations of metals and, therefore, lower tolerance to them (He et al. 2016). Chandrasekaran et al. (2015) demonstrated a positive correlation between Mg and Ca, but a negative correlation with metals such as Zn, Mn, and Ni when comparing these cations with specific metals, which is consistent with our results.

### Bacteria tolerance to heavy metals

The isolates *B. velezensis* C3-3 and *C. gottheilii* T106 exhibited growth at a concentration of 5 mM in all metals (Fig. 5), which represents a new resistance threshold (Abou-Shanab et al. 2007). The resistance to Cd in these isolates may be attributed to the presence of the *zntA* gene, which plays a role in intracellular homeostasis by facilitating Cd transport (Gallardo-Benavente et al. 2021). Furthermore, both isolates were found to possess a K+/H+/divalent ion antiporter, encoded by the *czcD* gene, which enables the efflux of Cd (II) and Co (II) from the cytoplasm (Moore et al. 2005). While both bacteria exhibited growth at 10 mM Co, strain C3-3 displayed higher sensitivity to Co-induced stress compared to T106. This difference could be explained by the absence of resistance-related genes from the *cbi* and *cob* clusters in the genome of C3-3, which were present in T106 (Abdullahi et al. 2021). The *cbiD*, *cbiE*, and *cbiG* genes, involved in cobalamin (vitamin B12) biosynthesis through early cobalt insertion, were absent in C3-3 (Balabanova et al. 2021).

T106, on the other hand, demonstrated greater growth in the presence of Co and Ni, which can be attributed to the presence of genes encoding ABCL-type transporter proteins, crucial for vacuolar sequestration of HMs (Khoudi 2021), and permease components *ddpA*, *ddpB*, *ddpC*, and *ddpD*, facilitating the uptake and reducing the toxicity of Co and Ni in the soil (Yun et al. 2020). Additionally, the presence of genes *cbiO*, *cbiM*, *cbiQ*, and *cbiN*, which constitute an ATP-dependent import system associated with increased Ni or Co uptake in bacteria such as *Propionibacterium*, *Ruegeria*, and *Kitasatospora*, has been reported (Yin et al. 2023). In contrast, C3-3 only exhibited the presence of genes *ddpA*, *ddpB*, *ddpC*, and *ddpD*, *ABC.PE.S*, *ABC.PE.P*, and *ABC.PE.P1* permeases, and substrate-binding proteins of the peptide/nickel transport system responsible for nickel stress tolerance (de Sosa et al. 2021).

Both isolates displayed resistance to 10 mM Mn. In C3-3, the *mntH* and *mntP* genes were identified, whereas T106 only possessed the *mntP* gene. These genes confer Mn resistance in *B. amyloliquefaciens* and *B. velezensis* by facilitating the expulsion of manganese ions at high intracellular concentrations (Luo et al. 2022). Furthermore, both genomes exhibited the presence of *mntA*, *mntB*, and *mntC* genes, encoding the ABC transporter responsible for capturing extracellular Mn ions in Mn-resistant *Bacillus anthracis* and *Staphylococcus aureus*.

## Conclusion

The bacterial communities isolated from RIs presented potential resistance and tolerance against HMs, favored by moisture and the presence of vegetation. Resistance and tolerance genes were identified in taxonomic groups such as Anaerolineales, Acidobacteria, and Proteobacteria. These genes exhibited a positive correlation with clay and silt and a negative correlation with sand, due to their ability to retain HMs, which could exert selective pressure on the presence of these genes. Resistance and tolerance were determined through various mechanisms of HMs detoxification, primarily mediated by enzymes such as oxidoreductases, metalloproteases, and hydrolases, as well as transmembrane proteins involved in the efflux of HMs out of the cell, such as efflux pumps and ion transmembrane transporters. The isolates *B. velezensis* C3-3 and *C. gottheilii* T106 were found to be tolerant to HMs such as Cd, Co, Mn, and Ni, mainly due to the presence of genes associated with ABC pumps, intracellular homeostasis for Cd transport, ion antiporter proteins (Cd and Co), cobalamin biosynthesis, ABCL-type transporter proteins, permease components for Co and Ni, ATP-dependent import system (Ni and Co), peptide/nickel transport system, efflux pumps (Mn), and transporters (Mn). Our results suggest that microbial communities from RIs have the ability to cope with HM, highlighting their potential in bioremediation processes in contaminated soils. Furthermore, these findings suggested that some environmental factors such as moisture and vegetation might shape bacterial resistance and tolerance to HMs.

### Supplementary Information

ESM 1 (PDF 426 kb)

## Data Availability

Raw sequencing reads were deposited in the NCBI Sequence Read Archive (SRA) under the project number PRJNA817243.
