# Supplementary material for: Metagenomic and genomic analysis of heavy metal-tolerant and -resistant bacteria in resource islands in a semi-arid zone of the Colombian Caribbean
Source: Environ Sci Pollut Res Int. 2023 Dec 21;31(4):5596–609. doi: 10.1007/s11356-023-30253-w (PMC10799150; doi:10.1007/s11356-023-30253-w)
Supplement: Supplementary file 1 — (PDF 426 kb) [file 11356_2023_30253_MOESM1_ESM.pdf]

**SUPPLEMENTARY MATERIAL**

**TABLE S1 Concentrations of HM found in the IRs during wet (W) and dry (D) seasons**

| **Season** | **Sample** | **Fe Available (mg/kg)** | **Mn Available (mg/kg)** | **Zn Available (mg/kg)** |
|---|---|---|---|---|
| D | Tru-1 | 27.36 | 23.53 | <1.00 |
| D | Tru-2 | 70.83 | 14.00 | 1.29 |
| D | Tru-3 | 65.82 | 28.04 | 2.01 |
| D | Tor-1 | 63.15 | 20.82 | 2.41 |
| D | Tor-2 | 49.38 | 19.72 | 1.72 |
| D | Tor-3 | 21.84 | 23.97 | 2.23 |
| D | Bra-1 | 10.88 | 7.79 | <1.00 |
| D | Bra-2 | 75.62 | 21.42 | 1.95 |
| D | Bra-3 | 46.28 | 26.12 | 2.91 |
| D | C-1 | 12.79 | 7.40 | <1.00 |
| D | C-2 | 10.40 | 8.41 | <1.00 |
| D | C-3 | 8.73 | 7.70 | <1.00 |
| W | Tru-1 | 17.38 | 2.3 | <1.00 |
| W | Tru-2 | 86 | 3.52 | 1.15 |
| W | Tru-3 | 45.82 | 3.38 | 1.2 |
| W | Tor-1 | 9.94 | 1.61 | 1.33 |
| W | Tor-2 | 5.81 | 1.19 | <1.00 |
| W | Tor-3 | 16.44 | 3.43 | 1.76 |
| W | Bra-1 | 13.7 | 2.37 | <1.00 |
| W | Bra-2 | 7.82 | 1.15 | <1.00 |
| W | Bra-3 | 9.64 | 1.26 | 1.32 |
| W | C-1 | 16.38 | 1.14 | <1.00 |
| W | C-2 | 6.15 | <1.00 | 1.02 |
| W | C-3 | 21.16 | 7.70 | <1.00 |

**Techniques:** Measured by Olsen solution extraction technique and quantified through atomic absorption spectrophotometry

**Cu Available:** <1.00 mg/kg
**Tru:** *Prosopis juliflora;* **Bra**: *Haematoxylum brasilet;* **Tor**: *Phitellobium dulce*
**C:** Control

**Table S2** Most abundant molecular functions in HM resistance genes in RIs during the dry (D) and wet (W) seasons under the presence (V) or absence of vegetation (C).

| Molecular Function (Metal) | Abundances (%) | Conditions | | | | P-value | | | |
|---|---|---|---|---|---|---|---|---|---|
| | | VW | CW | DV | DC | 1 | 2 | 3 | 4 |
| Copper-dependent protein binding(Cu) | 20.53% | 2332 | 5539 | 2467 | 3117 | | | | |
| Oxidoreductase(Cr, Mn, Co, Mo) | 20.52% | 3055 | 3855 | 2407 | 2216 | | +++ | +++ | * |
| Chromate tr. transporter(Cr) | 8.37% | 1969 | 2519 | 1891 | 2086 | | ++ | | |
| Manganese ion binding(Mn) | 7.87% | 1634 | 895 | 2216 | 1748 | | ++ | | |
| Hydrolase, Ligase (Co) | 6.46% | 1325 | 991 | 1123 | 1176 | * | ** | *** | |
| Cation tr. transporter (Co,Zn,Cd) | 6.3% | 1268 | 1210 | 1130 | 944 | | | | |
| Metallic ions tr. transporter(Mn/Zn) | 4.49% | 978 | 959 | 705 | 865 | | + | * | + |
| Ligase(Co) | 3.98% | 742 | 779 | 799 | 1024 | | +++ | +++ | |
| Hydrolase, Metalloprotease(Zn) | 2.66% | 712 | 1622 | 396 | 399 | | | +++ | |
| DNA binding(Cu, Mn, Ni/Co) | 2.61% | 741 | 689 | 589 | 589 | | | | |
| Active tr, transporter of antimonite(As) | 0.51% | 211 | 181 | 273 | 227 | | | | * |
| | **Final totals** | 20170 | 25135 | 17813 | 17954 | (*) 4 | 11 | 4 | 4 |
| | | | | | | (+) 0 | 5 | 4 | 1 |

**P Value:** 1: V y C (+, V>C); 2: W y D (+ D>W) ; 3: W y C -W y V (+,W y C>W y V) ; 4: D y V-D y C (+,D y C>D y V)
*:It indicates the opposite of what was previously stated
**significant differences:** ***/+++ p<0.001 **/++ p<0.01 */+ p<0.05
Tr : transmembrane

**Table S3** Most abundant molecular functions in HM tolerance genes in RIs during the dry (D) and wet (W) seasons under the presence (V) or absence of vegetation (C).

| Molecular Function (Metal) | Abundances (%) | CONDICIONS | | | | P Value | | | |
|---|---|---|---|---|---|---|---|---|---|
| | | VW | CW | DV | DC | 1 | 2 | 3 | 4 |
| Hydrolase / Protease(Zn) | 20.78% | 17377 | 12631 | 19874 | 15733 | + | * | * | |
| Peptide tr. transporter(Ni) | 18.69% | 16272 | 15333 | 15337 | 15627 | | | | |
| Hydrolase(Ni) | 17.13% | 15961 | 14002 | 13317 | 12864 | | + | | |
| ATP binding(Mn- Zn y Ni) | 15.25% | 12491 | 12782 | 13976 | 13842 | | + | | |
| Cations tr. transporter (Co-Zn-Cd) | 8.89% | 11821 | 13798 | 8612 | 7250 | | | | |
| Oxidoreductase(Co- Cr- Mn- Mo y Zn) | 5.06% | 4347 | 2794 | 4928 | 3398 | +++ | + | ** | ** |
| Ag ions tr. transporter(Ag) | 3.34% | 3426 | 2544 | 2361 | 2715 | | * | | |
| Methyl transferase(Co) | 2.00% | 1471 | 3532 | 1208 | 1914 | *** | | +++ | ++ |
| Ligase(Co) | 1.89% | 2932 | 1722 | 4312 | 3511 | | +++ | * | |
| Metal ion binding(Mn-Zn, Zn) | 0.95% | 1087 | 973 | 775 | 668 | | | | |
| | **Final totals** | 110750 | 107744 | 104673 | 99079 | (* )4 | 11 | 4 | 4 |
| | | | | | | (+) 0 | 5 | 4 | 1 |

**P Value:** 1: V y C (+, V>C); 2: W y D (+ D>W); 3: W y C -W y V(+, W y C>W y V); 4: D y V-D y C (+, D y C>D y V)
*****: It indicates the opposite of what was previously stated
**significant differences:** ***/+++ p<0.001   **/++ p<0.01   */+ p<0.05
Tr: transmembrane
